# Foot Posture Characteristics and Bilateral Load Distribution in African Male Recreational Runners: Insights from Foot Posture Index and 3D Scanning

**DOI:** 10.3390/jfmk10030361

**Published:** 2025-09-20

**Authors:** Yaasirah Mohomed Choonara, Glen James Paton

**Affiliations:** 1 Department of Podiatry, Faculty of Health Sciences, University of Johannesburg, Johannesburg 2006, South Africa; yaasirahc@uj.ac.za; 2 Department of Chiropractic, Faculty of Health Sciences, University of Johannesburg, Johannesburg 2006, South Africa

**Keywords:** lower limb biomechanics, foot morphology, clinical assessment, sports injury prevention, kinematics

## Abstract

**Background:** Recreational running is a globally popular activity known for its physical and mental health benefits, including stress reduction and improved quality of life. However, many recreational runners lack structured guidance, increasing their risk of lower limb injuries, often linked to variations in foot posture. Although African populations are well known for their endurance running abilities, limited research has examined their foot biomechanics and injury risk. This study addresses this gap by investigating foot posture and structure among African male recreational runners in South Africa. **Methods:** A cross-sectional, quantitative design was employed. Data were collected using structured data collection sheets, capturing demographic information, Foot Posture Index (FPI) scores, and Three-Dimensional (3D) foot scans. FPI provided a clinical evaluation of foot posture, while 3D foot scans delivered precise structural measurements. The aim was to describe and compare the foot posture characteristics and bilateral load distribution patterns in this population, using the Foot Posture Index (FPI) and 3D foot scanning as complementary assessment tools. **Results:** Findings showed agreement between FPI and 3D foot scan results, with both tools identifying a high prevalence of flexible flat feet among participants. Each method captured unique aspects of foot posture: FPI offered a qualitative overview, while 3D scans provided detailed, quantitative insights. This dual-assessment approach supports the value of using complementary methods in clinical and sports settings. **Conclusions:** This study suggests that integrating FPI and 3D scanning enhances the accuracy of foot posture assessments. Despite limitations such as a moderate sample size, the findings support personalized clinical interventions and footwear design tailored to the unique biomechanics of Black African male runners.

## 1. Introduction

Recreational running is a widely practised physical activity, celebrated for its numerous health benefits such as improved cardiovascular function, enhanced mental well-being, and stress reduction [1]. Despite these advantages, running-related injuries remain a persistent issue, with incidence rates ranging from 20% to 79% among recreational runners annually [2]. These injuries predominantly affect the lower extremities, often due to factors such as improper training regimens, poor footwear choices, and a lack of professional guidance or biomechanical assessment.

Foot posture and mechanics play a central role in both injury prevention and running performance. Over the past decades, various clinical and technological tools have been developed to evaluate foot structure and function. Among them, the Foot Posture Index (FPI-6) has gained widespread use in clinical and research settings due to its simplicity, cost-effectiveness, and ability to classify foot posture into pronated, neutral, or supinated categories. However, it is a subjective tool dependent on the clinician’s experience [1].

In contrast, advancements in digital technology have introduced three-dimensional (3D) foot scanning as a more precise and objective method for assessing foot morphology. These scans offer detailed insights into foot geometry, arch height, and plantar pressure distribution, allowing for a dynamic and quantifiable analysis. Several studies have compared FPI to 3D imaging modalities or weight-bearing CT to investigate their agreement and reliability, demonstrating that these methods can be complementary but also highlighting differences in precision and clinical interpretation [3,4].

However, most such comparative research has focused on European or North American populations, leaving questions about its generalizability to other groups with potentially different foot structures due to genetic, anatomical, or environmental factors. One underrepresented group is Black African male recreational runners, who, despite their strong presence in global athletics, have received limited study [3]. Their foot morphology may reflect genetic heritage, habitual barefoot activity, and culturally specific lifestyle patterns, making understanding their biomechanics important for injury prevention, footwear design, and rehabilitation strategies [3].

While previous studies have examined the relationship between FPI and advanced imaging in other populations [5,6], there remains a clear need to evaluate this relationship specifically in Black African male recreational runners. To address this gap, the present study examines the foot posture characteristics, bilateral load distribution, and prevalence of foot types in Black African male recreational runners, using both the FPI-6 and 3D foot scanning to provide complementary perspectives rather than a formal method comparison. By expanding the biomechanical knowledge base for this often-overlooked group, this research aims to support more inclusive, evidence-based clinical and sports practices.

## 2. Materials and Methods

### 2.1. Study Design and Population

This study employed a cross-sectional, quantitative design. A cross-sectional approach was chosen to analyse data collected at a single point in time from a specific population, allowing for an efficient assessment of foot posture characteristics and comparisons between measurement tools [7]. A retrospective framework was selected to optimize resource efficiency while enabling the analysis of a demographically specific cohort. This approach aligns with methodological recommendations for investigating structural biomechanical traits in targeted populations. The sample included 104 Black African male recreational runners aged 18–45 years. This age range was selected to encompass individuals at peak physical activity levels while avoiding the confounding effects of age-related degenerative or developmental changes in foot joints [8]. These participants were chosen to address the underrepresentation of this demographic in biomechanical research and to explore potential biomechanical differences relevant to their unique genetic, morphological, and lifestyle factors. Additionally, the Black African population affinity is the largest group within South Africa comprising approximately 51 million people [9]. A priori power analysis was conducted using G*Power software (GPower 3.1.9.7) to determine the required sample size for detecting medium effect sizes (f^2^ = 0.15) at a significance level of α = 0.05 and power (1 − β) = 0.80. The analysis indicated that a minimum of 92 participants was required. With a sample size of 104, the study met this requirement, ensuring sufficient power to detect significant associations.

### 2.2. Workflow Summary 

A streamlined workflow guided the study process:

1. Population Selection.
2. FPI-6 Assessment.
3. 3D Foot Scans.
4. Weight Measurement.
5. Statistical Analysis (McNemar, Pearson chi-square, Fisher’s exact test).

This structured methodology ensured a robust and comprehensive description of foot posture and load patterns using complementary assessment tools, aligning with the study’s objectives.

### 2.3. Participants

A total of 104 Black African male recreational runners aged 18–45 years were included. Participants were identified through institutional databases and selected based on predefined inclusion and exclusion criteria to ensure sample homogeneity. Inclusion criteria required self-reported recreational running activity, male sex, and identification with the Black African population affinity. Exclusion criteria included recent lower limb surgeries, musculoskeletal pathologies affecting gait or foot structure (e.g., bunions, neuromas), and any neurological disorders that could influence biomechanical outcomes. Data were also collected on running experience, including number of years running and average weekly training volume. These variables were statistically controlled for in the analysis to reduce their potential confounding effects on foot biomechanics.

### 2.4. Data Collection Methods

The data collection methods consisted of three aspects, namely Foot Posture Index (FPI-6), 3D barometric force plate foot scans, and weight measurements. These measurements were recorded on a Microsoft Excel^®^ spreadsheet.

1. Foot Posture Index (FPI-6):

The FPI-6 assessment used six criteria to evaluate foot posture [5]:

- Talar head palpation.
- Curvature of the lateral border.
- Calcaneal position in the frontal plane.
- Prominence of the medial longitudinal arch.
- Abduction/adduction of the forefoot.
- Extent of foot contact area.

Scores for each criterion ranged from −2 (indicative of supination) to +2 (indicative of pronation), resulting in a total score between −12 and +12. Foot posture was categorized as [5]:

- Neutral: −5 to +5
- Pronated: +6 to +12
- Supinated: −12 to −6

Two trained assessors independently evaluated each participant, with discrepancies resolved through consensus, ensuring consistency and reliability in FPI-6 scoring.

2. Three-Dimensional (3D) Foot Scans:

The Freemed force plate system captured foot biomechanics under static and dynamic conditions [10]. Participants stood with their feet shoulder-width apart, distributing weight evenly as the force plate recorded vertical forces, pressure distribution, and centre of pressure (CoP) data.

In addition to the static stance assessment, dynamic trials involving walking at a self-selected pace were conducted across the force plate. Each participant performed at least three valid walking trials, which were used to evaluate pressure distribution and CoP trajectory during gait. These data provided a detailed spatial analysis of foot structure and loading patterns, complementing the static FPI-6 assessments. Technical specifications of the Freemed system included high-resolution pressure sensors calibrated before each session. The accompanying software analysed the spatial and temporal aspects of foot biomechanics.

3. Weight Measurement:

Participant weight was recorded using a calibrated digital scale. This data was included to investigate potential correlations between body weight and foot posture, as weight influences load distribution and foot biomechanics. All data was captured on a structured Microsoft Excel^®^ spreadsheet used to record FPI-6 scores, 3D scan measurements, and participant weight. All data were anonymized and stored in a secure database. Missing or inconsistent entries were resolved by cross-referencing original records and seeking consensus between researchers.

### 2.5. Data Analysis

To ensure measurement reliability, both intra- and inter-rater reliability were assessed. Inter-rater reliability, measuring consistency between raters, was evaluated by comparing two independent assessors’ analyses of CoP trajectory data using Cohen’s kappa statistic, which showed substantial agreement (κ > 0.633). Intra-rater reliability, assessing consistency within the same rater over time, was tested by reanalysing a subset of data after two weeks, yielding excellent reproducibility (κ > 0.85). Analyses were performed using IBM SPSS Statistics Package 28, with results presented in cross-tabulations and Chi-square (or Fisher’s exact test). The *p*-values was set at 0.05 for significant findings. Quantitative statistical methods were employed to assess the agreement and relationships between FPI-6 scores and 3D foot scan measurements. The following tests were selected for their ability to analyse categorical and ordinal data effectively:

The McNemar test was employed to evaluate paired categorical data, assessing whether significant differences existed in foot posture classifications between the FPI-6 and 3D foot scans, chosen for its appropriateness in analyzing paired diagnostic outcomes [11]. The Pearson chi-square test was used to examine associations between categorical variables, such as foot posture categories, and was suitable for large sample sizes, provided that at least 80% of the cells had expected frequencies greater than five [12]. When these assumptions were not met, Fisher’s exact test was utilized to provide accurate *p*-values for smaller samples, ensuring the statistical robustness of the findings [12].

### 2.6. Limitations of Methods

FPI-6 may introduce variability due to its subjective nature, although steps were taken to minimize this by employing trained assessors and resolving discrepancies through consensus [13].

The 3D foot scans, while highly detailed, may not fully capture biomechanical variations during running, as assessments were performed under static and controlled dynamic conditions [14].

### 2.7. Data Recording and Reliability

All data were anonymized and organized in Microsoft Excel^®^. Statistical analyses were performed in IBM SPSS Statistics (v28.0). Intra- and inter-rater reliability for FPI and pressure data were assessed using Cohen’s Kappa coefficients (κ = 0.633–0.85). Appropriate statistical tests, including McNemar’s test, Pearson’s chi-square, and Fisher’s exact test, were applied based on data type and sample size.

This structured methodological framework ensured robust internal validity while allowing the data to address the study’s core objectives, namely, evaluating congruence between assessment tools and identifying biomechanical traits in a demographically specific athletic population.

## 3. Results

Cross Tabulations were performed to compare the FPI, foot type, centre of pressure (CoP) and load percentages of the right feet and left feet, and to determine the association between foot types, CoP, FPI and load percentages. The McNemar, Fisher’s Exact, Pearson Chi-Square tests were performed to understand the differences and associations between the above-mentioned variables.

### Statistical Tests

**Figure 1:**
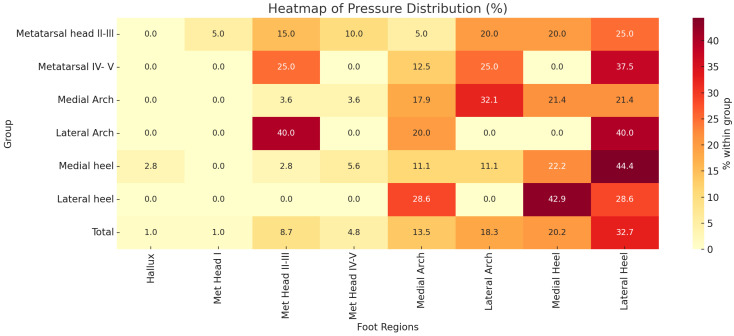
Heatmap of pressure distribution (%) across foot regions (*x*-axis) by originating foot regions (*y*-axis). Warmer colours indicate higher cross-region pressure contributions. Note: Hallux and Met Head I regions are excluded from the y-axis because they showed minimal or inconsistent cross-region pressure transfer in our data, reflecting their typically localized loading patterns.

Statistical analysis revealed several associations between foot posture, pressure distribution, and weight. A significant relationship was found between the centre of pressure and the side of the foot using the McNemar test (*p* = 0.021), suggesting asymmetry in pressure distribution. However, no significant differences were observed between the right and left Foot Posture Index (FPI), with McNemar’s test yielding a *p*-value of 1.000. Fisher’s exact test indicated no significant association between rigid foot type and medial pressure location (*p* = 0.397). A significant association was observed between arch and foot region distribution (*p* = 0.038), with Figure 1 confirming this relationship (*p* < 0.001). No significant differences were found between flexible and rigid foot types when comparing pressure regions (*p* = 0.463), nor between FPI and arch region (*p* = 0.977) or medial-lateral pressure distribution (*p* = 1.000). The cross-tabulation of FPI left and right (RQ10) also showed no significant difference (*p* = 0.968 and 0.986, respectively). Additionally, no significant associations were found between weight and FPI (*p* = 0.448) or between weight and specific foot regions, including hallux, metatarsals, arch, and heel (*p* = 0.535).

## 4. Discussion

The findings of this study demonstrate notable variability in foot posture and load distribution among runners. While most participants exhibited flexible feet with generally normal alignment, a significant proportion displayed pronation, and bilateral differences were observed. The presence of asymmetry between right and left feet suggests that foot biomechanics may not be uniform across sides, raising implications for running performance and injury risk. Functional dominance and structural disparities may contribute to these differences, potentially altering load distribution and stability [15,16]. Three-dimensional foot scan data supported these observations, revealing side-specific variations in centre of pressure and load distribution. Significant differences between feet underscore the lack of bilateral symmetry even among runners with similar foot types. Together, these findings highlight the importance of evaluating each foot individually during clinical and performance assessments to account for asymmetrical biomechanical patterns [17].

### 4.1. Agreement or Discrepancies Between FPI-6 and 3D Foot Scans

**Figure 2:**
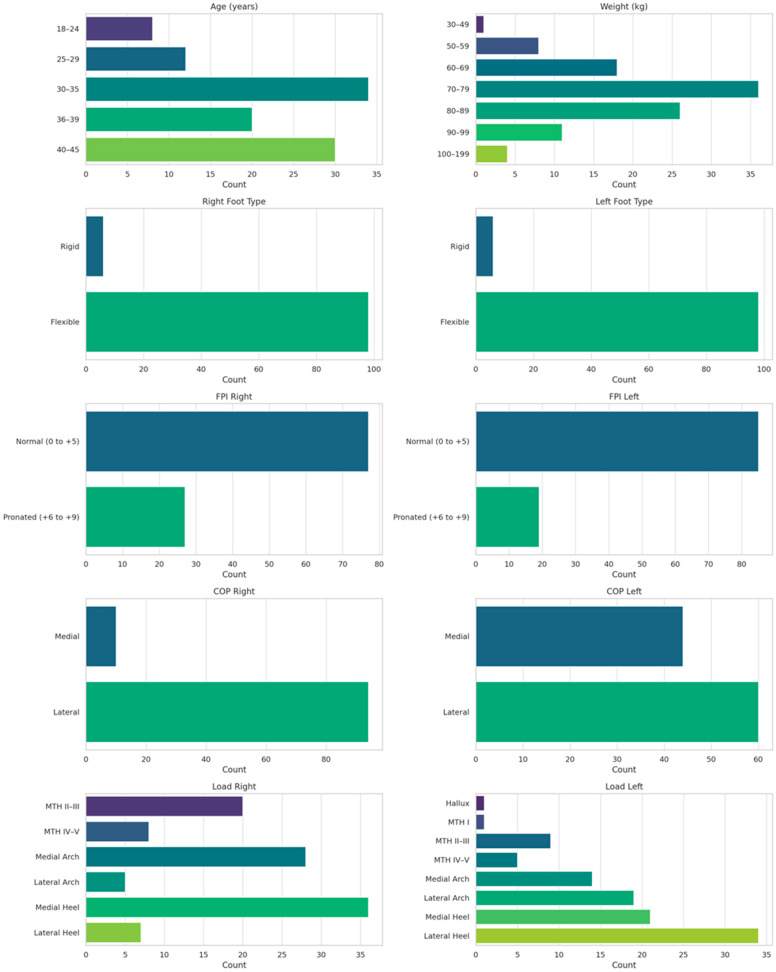
Demographic and frequency summary of participants: age, weight, foot type (right and left), Foot Posture Index (FPI: right and left), Centre of pressure (CoP), Foot loading (right and left).

**Figure 3:**
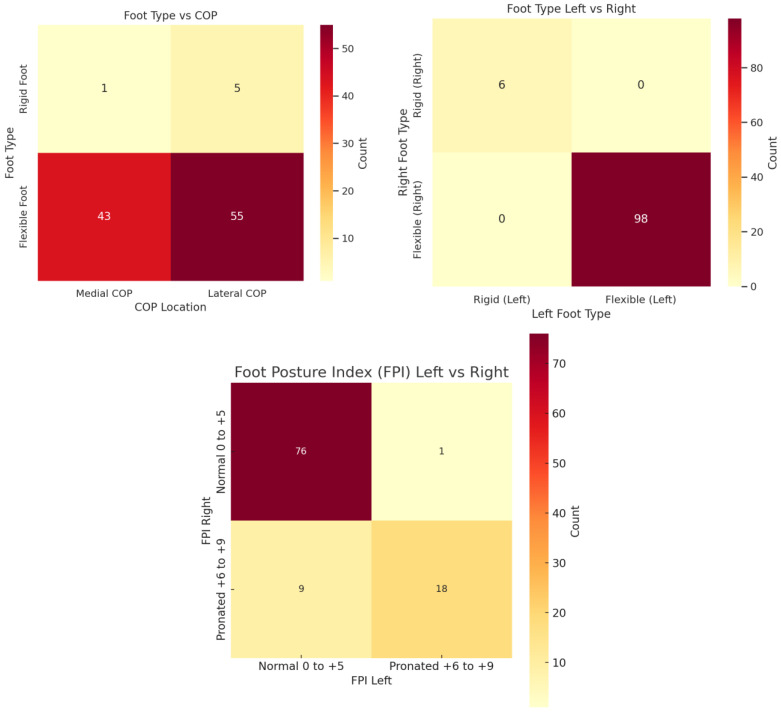
Heatmaps demonstrating the variables within this study. **Top left**: Foot type and centre of pressure. **Top right**: foot type vs. foot flexibility. **Bottom**: left foot posture index vs. right foot posture index.

**Figure 4:**
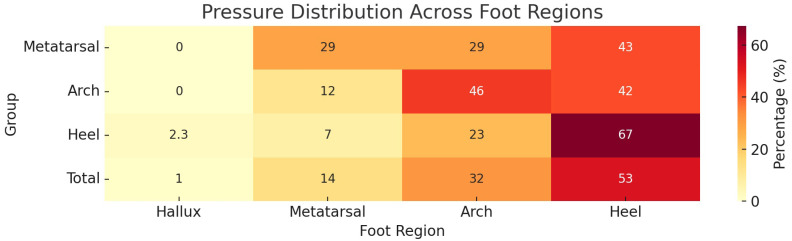
Heatmap visualizing the pressure distribution across foot regions (Hallux, Metatarsal, Arch, and Heel) for each group. The warmer colours represent higher percentages.

The FPI results reveal a diverse spectrum of foot postures among the participants (Figure 2), with six runners displaying rigid flat feet and 98 classified as having flexible feet. For the right foot, 77 participants showed normal alignment, while 27 exhibited pronation. Similarly, on the left foot, 85 participants had normal posture, with 19 displaying pronation. Notably, a majority maintained consistent posture across both feet, but significant differences between right and left foot postures (McNemar test, *p* = 0.021) suggest that foot biomechanics may differ bilaterally among runners (Figure 3, bottom heatmap). Significant differences in load distribution between feet (McNemar test *p* > 0.001, Fisher’s Exact test *p* = 0.038) support the notion that runners adapt to unique biomechanical patterns, bearing asymmetrical loads influenced by stride, gait, or structural variations [18,19]. Figure 4 demonstrates the findings by revealing variations in the centre of pressure and load distribution between feet. 

The descriptive assessment using complementary tools between the FPI-6 and 3D foot scans (Figure 1 and Figure 3) aimed to identify whether these tools align in assessing foot biomechanics. Notable alignment between the methods suggests that both tools capture complementary aspects of foot posture. Fisher’s Exact test results showed no significant differences in centre of pressure patterns between rigid and flexible feet, challenging the assumption that rigidity or flexibility inherently dictates pressure distribution (Figure 3, top right). Although the statistical findings did not reveal significant associations between foot type (rigid or flexible) and pressure distribution, this absence of significance is informative in itself. It suggests that while both the FPI-6 and 3D foot scans are effective in capturing structural and pressure-related data respectively, one tool may not directly substitute for the other. The lack of congruent findings between them does not invalidate either tool but rather supports the notion that each assesses different aspects of foot function, with FPI-6 focusing on observable alignment and morphology, and the 3D scans capturing functional pressure patterns. These non-significant results highlight a critical methodological insight: static posture assessments (FPI-6) may not reliably predict dynamic loading behaviours captured by 3D pressure scans. This divergence emphasizes the importance of using both tools in a complementary manner rather than relying on one as a standalone diagnostic method.

Similarly, no significant differences in centre of pressure were observed between normal and pronated foot postures. This lack of association highlights the complexity of load dynamics, suggesting that static foot posture categories may not reliably predict pressure distribution or injury risk [20]. Therefore, the absence of significant relationships reinforces the clinical value of integrating multiple assessment tools to gain a holistic picture of foot biomechanics. While FPI-6 provides a quick and accessible structural snapshot, the 3D scan’s strength lies in its ability to detect nuanced pressure variations that may not correspond neatly to static classifications. These findings point toward the need for a more holistic approach to foot biomechanics, integrating individual variations in alignment, muscle activation, and movement patterns to better understand and address injury mechanisms [21].

While the FPI-6 is effective for initial classification and qualitative assessment, the 3D foot scan-derived pressure heatmaps (Figure 1 and Figure 4) provide more granular insight into how load is distributed across foot regions. For instance, Figure 1 illustrates cross-region pressure transfer patterns, while Figure 4 visualizes direct regional loading differences between groups. Together, these tools can provide a fuller understanding of foot posture by combining the FPI’s accessibility with the 3D scan’s detailed insights. However, incorporating dynamic assessments such as gait analysis would further enrich the interpretation, as static tools alone cannot capture the complexities of running biomechanics.

### 4.2. Practical Implications

The findings (Figure 1, Figure 2, Figure 3 and Figure 4) underscore the importance of bilateral assessments in clinical and sports settings. By identifying asymmetries in foot posture and pressure distribution, practitioners can tailor interventions to address specific biomechanical needs [22]. For instance, targeted strength and mobility exercises can help mitigate side-dominance effects and promote balanced biomechanics, reducing the risk of injury [23]. Furthermore, the insights gained from this study could guide footwear design, encouraging manufacturers to develop adaptive shoes that respond to individual pressure patterns rather than rigidly adhering to static foot types [24].

### 4.3. Limitations of the Study

While the FPI-6 offers a quick, cost-effective means of evaluating foot posture, its subjective nature and reliance on static assessments limit its reliability and comprehensiveness. Variability in assessor experience can further compromise the consistency of results. On the other hand, 3D foot scans provide highly accurate and objective measurements but remain inaccessible in many settings due to cost and technical expertise requirements. Like the FPI-6, 3D scans also lack the ability to capture dynamic foot behaviours, which are critical during running. Integrating both tools with dynamic assessments such as gait analysis would address these limitations, providing a more holistic view of foot function and biomechanics [20]. However, in this study, we were limited to performing gait analysis rather than running analysis, as the software used did not support the extraction of dynamic data specific to running. This constrained our ability to confirm the observed asymmetries under more functionally relevant, high-impact conditions.

### 4.4. Future Research Directions

This study reveals key opportunities for future investigations aimed at expanding the biomechanical understanding of foot posture in Black African male runners. Primarily, future research should prioritize dynamic, real-time methodologies such as three-dimensional gait analysis, in-shoe plantar pressure mapping, and electromyographic (EMG) analysis to capture neuromuscular coordination and kinetic variability during running [14,20,21]. These approaches would overcome the inherent limitations of static assessments, providing more ecologically valid data on the interaction between foot structure and movement patterns. Specifically, future studies should incorporate advanced dynamic assessment tools, such as gait laboratories or wearable plantar pressure systems during running, to confirm asymmetries and loading behaviours under functional, high-impact conditions. These tools would strengthen the biomechanical claims and ensure more accurate screening and intervention development.

Longitudinal studies are also essential to assess how foot posture and load distribution evolve over time and in response to various interventions, including strength training, footwear modification, and orthotic usage [8,17]. By tracking biomechanical adaptations across different phases of athletic activity, such studies could elucidate causal relationships between postural abnormalities and injury risk.

Another critical avenue involves the development of hybrid assessment models. Machine learning algorithms trained on high-resolution foot scan data, and FPI-6 scores could enhance predictive analytics and diagnostic precision, particularly in low-resource settings where comprehensive equipment is not available [22]. Cross-sectional studies incorporating additional sociodemographic variables, including occupation, barefoot walking frequency, and habitual footwear usage, could further contextualize biomechanical variations within specific cultural and environmental frameworks [22].

Finally, the role of personalized biomechanics in precision sports medicine and preventive care warrants exploration. Future work should investigate how individualized foot posture profiles can be integrated into custom rehabilitation protocols, targeted exercise regimens, and responsive orthotic and footwear technologies [23,24]. These innovations could drive more effective injury prevention and athletic performance enhancement, particularly in populations underrepresented in current biomechanical research.

### 4.5. Potential Applications of the Study

The implications of this study span multiple domains including clinical practice, athletic performance, public health, and commercial product design. Clinicians in podiatry and sports medicine can use the combined insights from the FPI-6 and 3D foot scan to improve diagnostic accuracy and treatment planning. For example, early identification of bilateral asymmetries and atypical loading patterns can guide interventions to correct biomechanical inefficiencies and reduce the risk of chronic injuries such as plantar fasciitis and medial tibial stress syndrome [2,19].

In athletic contexts, coaches and physiotherapists can incorporate these findings into screening protocols to monitor postural alignment and load distribution symmetry. Training regimens can then be tailored to address identified deficits, improving performance and reducing injury susceptibility [15,16]. Such personalized approaches are especially beneficial for runners, where repetitive stress and ground contact time amplify the impact of even minor asymmetries.

Furthermore, the study’s outcomes have implications for the design and development of footwear and orthotic devices. Shoe manufacturers can utilize population-specific data to design adaptive footwear with variable cushioning zones and ergonomic structures tailored to foot posture and pressure distribution profiles [12,25]. These findings can also inform orthotic prescription practices by identifying structural imbalances that require correction, allowing clinicians to recommend custom orthoses that address asymmetries in foot alignment and load bearing. For African runners in particular, integrating such data into footwear design and orthotic prescription may enhance comfort, performance, and injury prevention, by accounting for population-specific anatomical and biomechanical characteristics that are often overlooked in existing designs. In underrepresented populations such as African male recreational runners, region-specific anatomical data can help rectify biases in current footwear design paradigms.

From a public health perspective, incorporating foot posture screening into community health initiatives may support injury prevention strategies and encourage long-term participation in physical activity [1]. Moreover, the study serves as a resource for healthcare education, offering biomechanical benchmarks that can inform clinical training programs in physiotherapy, podiatry, and rehabilitation sciences.

## 5. Conclusions

This study highlights the strengths and limitations of the FPI-6 and 3D foot scans in assessing foot posture among African male recreational runners. While both tools provide valuable insights, they capture distinct and complementary aspects of foot biomechanics. The FPI-6 offers a simple, accessible method for initial classification but is limited by its reliance on static evaluation and subjective interpretation. In contrast, 3D foot scans deliver precise, quantitative data on foot structure and load distribution, though they, too, are constrained by their static nature and resource demands.

The observed bilateral differences in foot posture and pressure distribution underscore the complexity of individual biomechanics, challenging traditional assumptions about foot type and pressure distribution. These findings emphasize the need for a more dynamic, personalized approach to foot assessment. Integrating static tools like the FPI-6 and 3D scans with dynamic gait analysis could provide a more comprehensive understanding of foot biomechanics, informing targeted interventions, injury prevention strategies, and the design of adaptive footwear. Future research should address the study’s limitations by expanding the sample size, incorporating longitudinal designs, and examining the interplay between foot posture, environmental factors, and running performance. Refining the complementary roles of the FPI-6 and 3D foot scans could pave the way for a more holistic, evidence-based approach to assessing and optimizing foot biomechanics in both clinical and athletic populations.

## Data Availability

Data are available on request from the corresponding author.

## Abbreviations

The following abbreviations are used in this manuscript:

3D	Three-dimensional
FPI	Foot Posture Index
